# Supplementary figures and images for: Action mechanism of a novel agrichemical quinofumelin against Fusarium graminearum
Source: eLife. 2025 Aug 20;14:RP105892. doi: 10.7554/eLife.105892 (PMC12367298; doi:10.7554/eLife.105892)

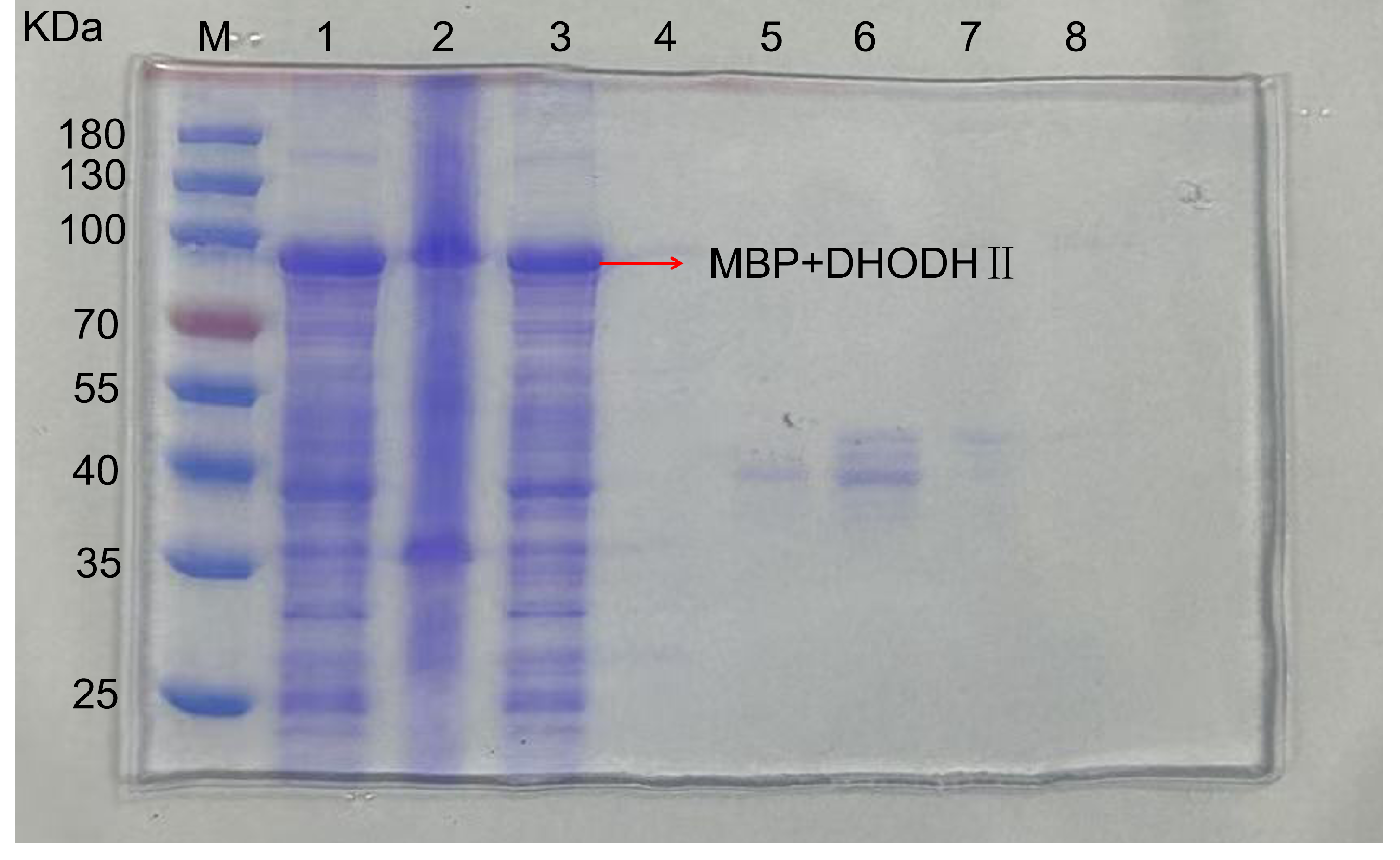

Supplement: Figure 6—figure supplement 1—source data 1. [file elife-105892-fig6-figsupp1-data1.zip › Figure 6-figure supplement 6-source data 1. TIFF file containing original western blots for Figure S6A, indicating the relevant bands and treatments/a.tif]

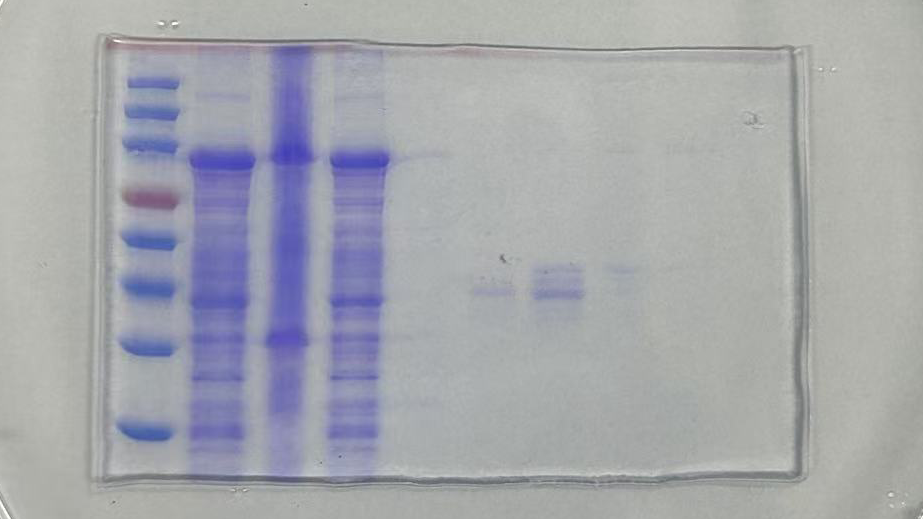

Supplement: Figure 6—figure supplement 1—source data 2. [file elife-105892-fig6-figsupp1-data2.zip › Figure 6-figure supplement 6-source data 2. Original files for SDS-PAGE analysis displayed in Figure S6A/a.tif]

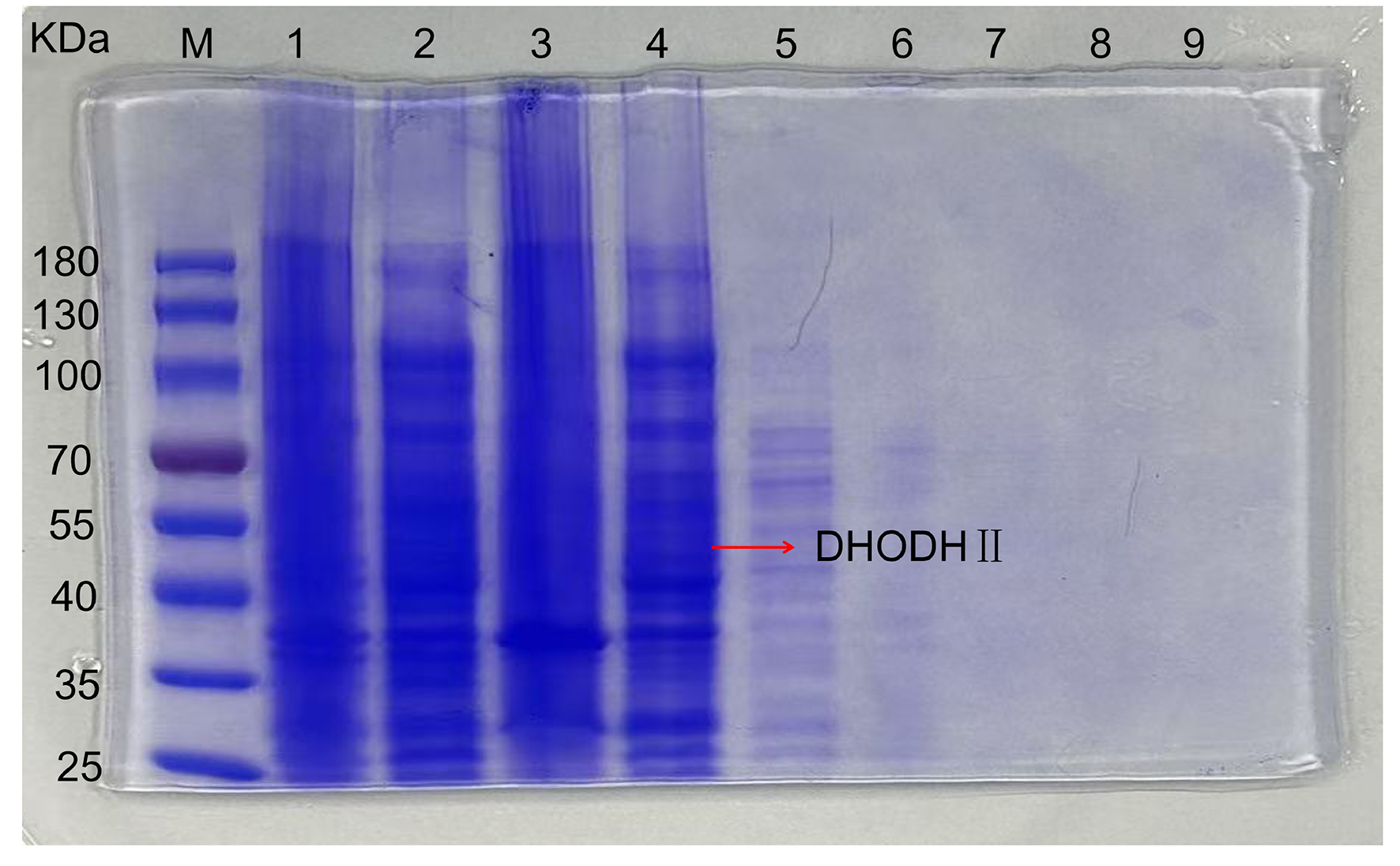

Supplement: Figure 6—figure supplement 1—source data 3. [file elife-105892-fig6-figsupp1-data3.zip › Figure 6-figure supplement 6-source data 3. TIFF file containing original western blots for Figure S6b, indicating the relevant bands and treatments/b.tif]

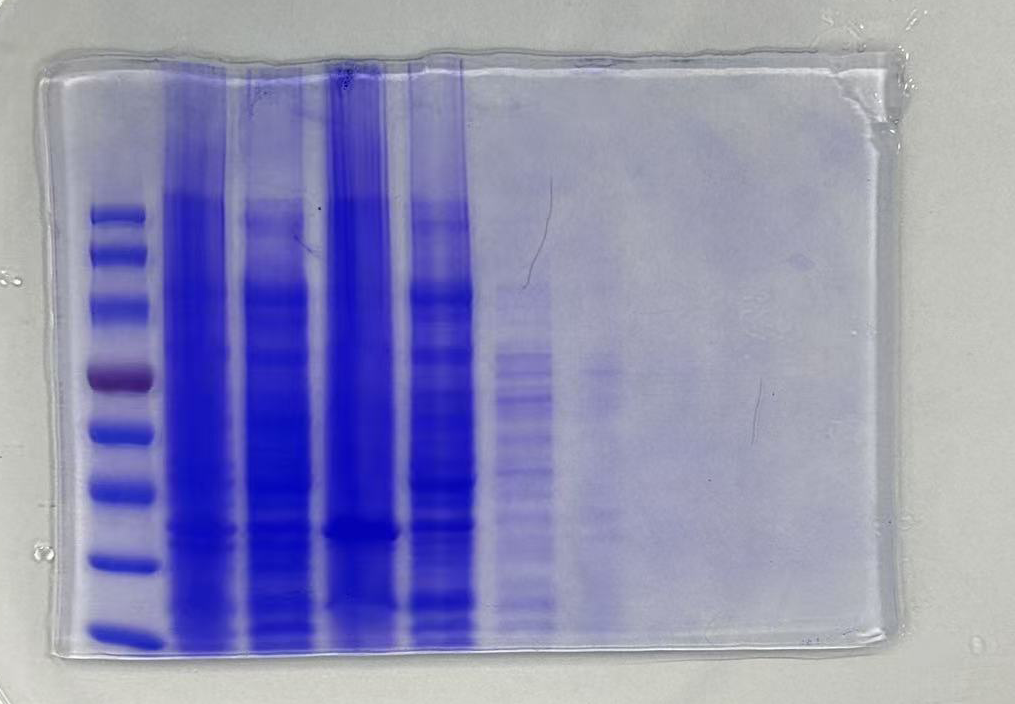

Supplement: Figure 6—figure supplement 1—source data 4. [file elife-105892-fig6-figsupp1-data4.zip › Figure 6-figure supplement 6-source data 4 Original files for SDS-PAGE analysis displayed in Figure S6b/b.tif]

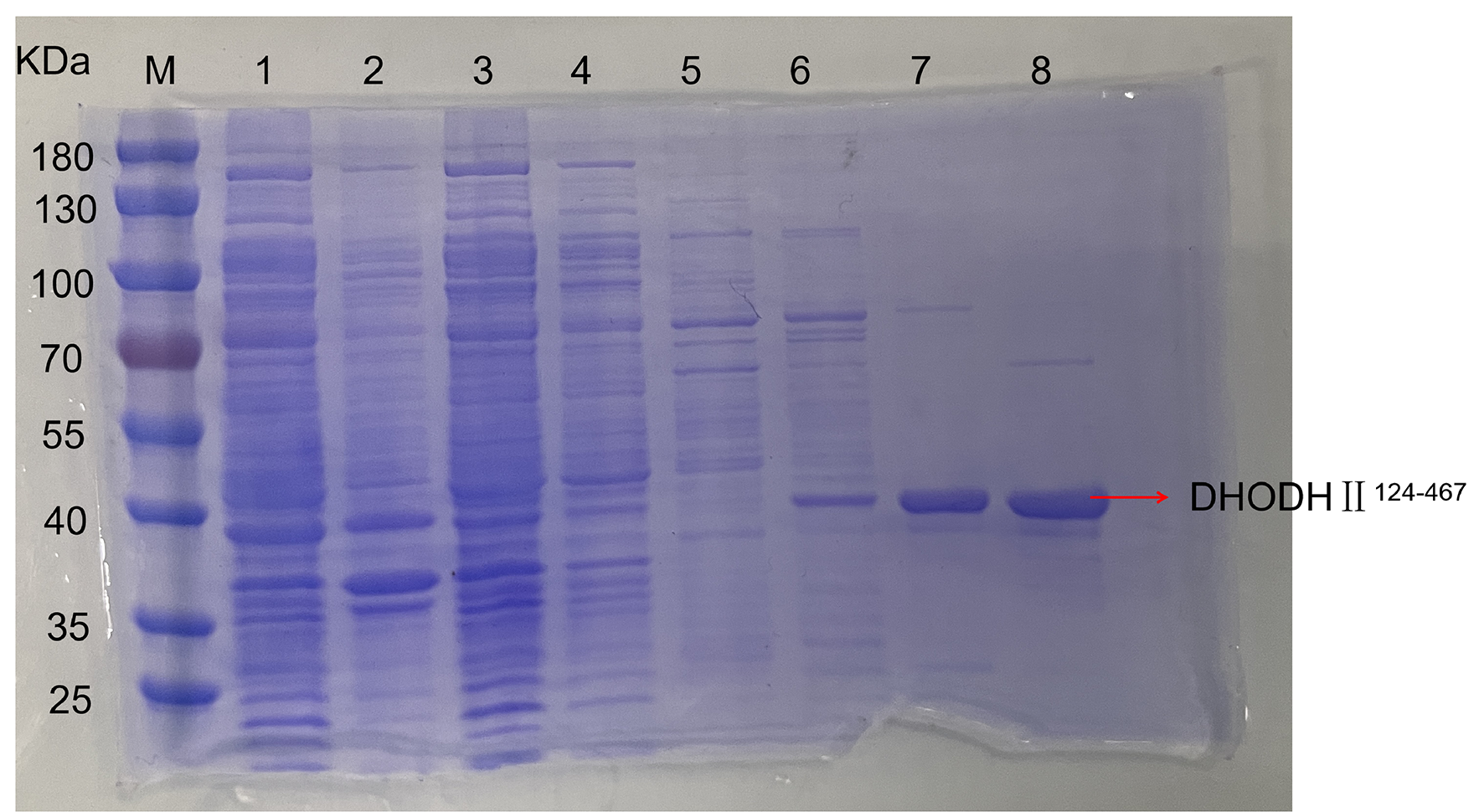

Supplement: Figure 6—figure supplement 1—source data 5. [file elife-105892-fig6-figsupp1-data5.zip › Figure 6-figure supplement 6-source data 5. TIFF file containing original SDS-PAGE for Figure S6c, indicating the relevant bands and treatments/c.tif]

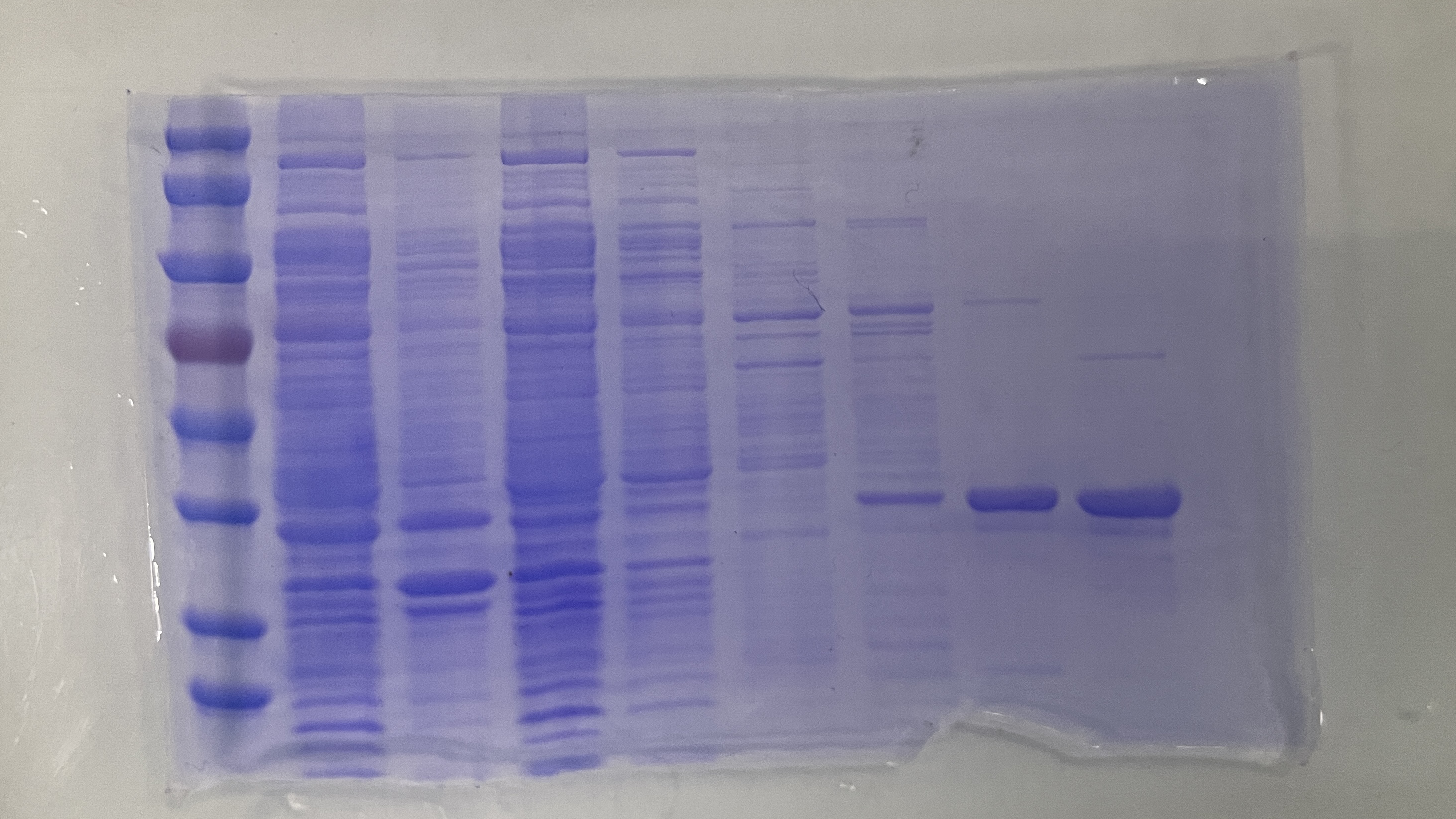

Supplement: Figure 6—figure supplement 1—source data 6. [file elife-105892-fig6-figsupp1-data6.zip › Figure 6-figure supplement 6-source data 6. Original files for SDS-PAGE analysis displayed in Figure S6c/c.tif]
