# Supplementary material for: Action mechanism of a novel agrichemical quinofumelin against Fusarium graminearum
Source: eLife. 2025 Aug 20;14:RP105892. doi: 10.7554/eLife.105892 (PMC12367298; doi:10.7554/eLife.105892)
Supplement: Supplementary file 1. [file elife-105892-supp1.docx]

Supplementary information

**Action mechanism of a novel agrichemical quinofumelin against** ***Fusarium graminearum***

Qian Xiu ^a,1^, Xiaoru Yin ^a,1^, Yuanyuan Chen ^b,1^ Ziyang Zhang ^a^, Yushuai Mao ^a^, Tianshi Wang ^b^, Jie Zhang ^a^, Mingguo Zhou ^a,2^, Yabing Duan ^a,2^

^a^ College of Plant Protection, State Key Laboratory of Agricultural and Forestry Biosecurity, Nanjing Agricultural University, Nanjing, 210095, China.

^b^ College of Science, Nanjing Agricultural University, Nanjing, 210095, China.

^1^ Qian Xiu, Xiaoru Yin and Yuanyuan Chen contributed equally to this work.

^2^ Corresponding author: E-mail address: mgzhou@njau.edu.cn (Mingguo Zhou); dyb@njau.edu.cn (Yabing Duan)

**Table S1. GO analysis of down- and up-regulated DEGs.**

| Description | Up | Down |
| --- | --- | --- |
| Vitamin binding | 0 | 5 |
| Transition metal ion binding | 3 | 10 |
| Tetrapyrrole binding | 3 | 3 |
| RNA biosynthetic process | 2 | 4 |
| Phosphopantetheine binding | 0 | 4 |
| Peroxidase activity | 1 | 1 |
| Oxidoreductase activity | 1 | 11 |
| Nucleic acid-templated transcription | 4 | 8 |
| NADP binding | 0 | 3 |
| Monooxygenase activity | 1 | 6 |
| Monocarboxylic acid metabolic process | 1 | 2 |
| Modified amino acid binding | 0 | 4 |
| Homeostatic process | 1 | 1 |
| Heme binding | 3 | 3 |
| Chemical homeostasis | 1 | 1 |
| Cellular amino acid catabolic process | 0 | 2 |
| Amide binding | 0 | 4 |

**Table S2. KEGG analysis of down- and up-regulated DEGs.**

| Description | Up | Down |
| --- | --- | --- |
| Tryptophan metabolism | 1 | 2 |
| Thiamine metabolism | 2 | 0 |
| Pantothenate and CoA biosynthesis | 0 | 2 |
| Nitrogen metabolism | 0 | 2 |
| Biosynthesis of nucleotide sugars | 2 | 0 |
| Amino sugar and nucleotide sugar metabolism | 3 | 0 |
